# What Do We Know about Anthracofibrosis? A Literature Review

**Published:** 2017

**Authors:** Hamdireza Jamaati, Amirsina Sharifi, Maryam Sadat Mirenayat, Majid Mirsadraee, Kazem Amoli, Hassan Heidarnazhad, Naseh Sigari, Kayvan Saeedfar, Shahram Kahkouee, Mihan Pourabdollah Toutkaboni, Esmaeel Mortaz, Seyed Mohammadreza Hashemian, Abdolreza Mohamadnia

**Affiliations:** 1 Chronic Respiratory Diseases Research Center, National Research Institute of Tuberculosis and Lung Diseases (NRITLD), Shahid Beheshti University of Medical Sciences, Tehran, Iran; 2 Students’ Scientific Research Center (SSRC), Tehran University of Medical Sciences, Tehran, Iran; 3 Department of Internal Medicine, Faculty of Medicine, Islamic Azad University-Mashhad Branch, Mashhad, Iran; 4 Department of Internal Medicine (Pulmonary), Tehran University of Medical Sciences, Tehran, Iran; 5 Internal Medicine Department, Faculty of Medicine, Kurdistan University of Medical Sciences, Sanandaj, Iran; 6 Mycobacteriology Research Center, NRITLD, Shahid Beheshti University of Medical Sciences, Tehran, Iran; 7 Pediatric Respiratory Diseases Research Center, NRITLD, Shahid Beheshti University of Medical Sciences, Tehran, Iran; 8 Virology Research Center, NRITLD, Shahid Beheshti University of Medical Sciences, Tehran, Iran

**Keywords:** Anthracosis, Anthracofibrosis, Anthracotic bronchitis, Biomass fuel, Pulmonary tuberculosis

## Abstract

Recently, the significance of anthracosis in the tracheobronchial tree, lung parenchyma, and even non-respiratory organs has been postulated and discussed in association with other diseases, especially tuberculosis.

We reviewed the current literature by using the following key words in Medline/PubMed, Embase, and Google Scholar databases: anthracosis, anthracofibrosis, anthracotic bronchitis, biomass fuels, and mixed-dust pneumoconiosis. The bibliographies of eligible papers were also reviewed for further relevant articles. A total of 37 studies were assessed. The content of these studies was then divided into specific categories.

Considering the pathogenesis, along with histopathological, radiological, and bronchoscopic results regarding anthracotic lesions, we suggest these findings be defined as “ANTHRACOSIS SYNDROME”. For the first time, we describe a syndrome involving black pigmentation, which was previously thought to involve only the tracheobronchial tree. Until recently, it was not considered to be a single syndrome with different sites of involvement.

## INTRODUCTION

Anthracosis is a term used to describe black pigmentation of the tracheobronchial tree involving both mucosal and submucosal layers and lung parenchyma, or black pigmentation in macrophages caused by the deposit of carbon, silica, and quartz particles (1–4). Thus, it is not a disorder, per se (5). It was first defined by Cohen in 1951 (6). If anthracosis is associated with mucosal proliferation resulting in luminal obliteration and/or obstruction, it refers to the condition anthracofibrosis, which was first described by Chung et al. in 1998 (7). There have been other synonyms for these conditions, including anthracotic bronchitis, anthracostenosis that first used by Törün et al*.*(8) specifically for patients with a history of long-term exposure to biomass smoke(9), and bronchial anthracosis(10). Given the widespread clinical manifestations and the histopathological, radiological, and bronchoscopic findings discussed in this study, we assume that the black pigmentations would cause deposits to form in multiple organs. Thus, it is more appropriate to consider this condition “ANTHRACOSIS SYNDROME”.

Anthracosis is an ancient disease, reported even in mummies (11). However, recently, there has been an interest in recognizing its clinical significance, as anthracosis is often an incidental finding during bronchoscopic evaluation of patients with non-specific clinical symptoms such as cough, dyspnea, phlegm, and wheezing (12). On the other hand, anthracofibrosis may be indicative of a chronic disease of both the tracheobronchial tree and lung (13).

Here, we reviewed the current information on anthracosis and anthracofibrosis, including epidemiology, etiology, pathogenesis, clinical manifestation, and natural course, associated conditions, mediastinal lymphadenopathy, cancer susceptibility, lung parenchymal involvement, bronchopneumonia susceptibility, other organ involvement, diagnosis method, and finally, prevention and treatment.

### Data Sources

We reviewed the current literature using the following key words in Medline/PubMed, EMBASE, and Google Scholar databases: anthracosis, anthracofibrosis, anthracotic bronchitis, biomass fuels, and mixed-dust pneumoconiosis. The bibliographies of eligible papers were also reviewed for further relevant articles. The institutional review board of Shahid Beheshti University of Medical Sciences approved our study protocol. Finally, 37 manuscripts most relevant to our objective, evaluating different aspects of “ANTHRACOSIS SYNDROME”, from epidemiology and clinical manifestation to etiology and pathogenesis, were reviewed by five independent reviewers.

### Epidemiology:

Anthracofibrosis was not reported in Europe until 2008, and no cases had been reported in Spain until 2012(14–16). In contrast, the condition is more frequently found in rural areas of Asia, especially in Middle East and Far East countries.

Epidemiological data on anthracofibrosis are limited. Most researchers have reported a female preponderance, with a median age of 67.9 years (ranging from 21 to 97 years) (7, 15, 17–19).

Although chronic airway disease in relation to indoor pollution has been found in many countries including Nepal, China, India, Saudi Arabia, Mozambique, Columbia, Guatemala, Bangladesh, Turkey, Greece, and Indonesia, most cases of anthracofibrosis have been reported from Iran and Korea(20).

An Iranian study, with the largest number of participants (*n* = 778) who had both bronchial anthracosis and/or anthracofibrosis, found that the majority of female patients were housewives, whereas most of the male patients were farmers or manual workers (21). Distribution of anthracofibrosis prevalence is not identical in different parts of Iran. Although Mashhad, Zahedan, Tabriz, Kerman, and Tehran (all capital cities of a province) have reported various cases of anthracofibrosis, there has been no report of the disease in Fars province. This finding is more valuable when we consider that the residents of Fars province are in contact with all the same factors that might cause the disease, owing to the similar lifestyle and exposure to fossil fuel emission in different rural areas(22).

Epidemiological studies in Korea have shown that anthracofibrosis is usually found in three groups: first, in patients with chronic obstructive pulmonary disease (COPD) who did not respond well to the usual treatments; second, in patients with bronchial asthma with no clinical response to standard treatments; and third, in patients with abnormal radiographic findings but who do not display symptoms(18, 23–25). In both Korean and Iranian rural homes, the kitchen is a place for cooking and heating using biomass fuels such as wood, leaves, and crop residues. In this setting, ventilation is almost always restricted to a single window on the roof, resulting in inadequate air circulation. This housing condition and lifestyle are often the same in studies reporting BAF in other countries(18). Previous studies have revealed that cooking smoke is associated with a higher risk of chronic lung diseases. Although there are limited data on patients with BAF in developed countries, some cases of BAF have been reported. These patients were immigrants to North America from developing countries such as India, where biomass fuel had been used(18, 26, 27).

A study published in 2010 reported that BAF with associated pulmonary tuberculosis (PTB) was common in immigrants to Canada from the Indian subcontinent(28). In this study involving 61 patients of foreign origin with PTB, 10 (16.7%) were diagnosed with BAF; nine of these patients were from the Indian subcontinent and one was from Vietnam. As with previous studies, the majority of patients were females, with a mean age of 69.8 ± 9.3 years(15).

These data suggest that most patients diagnosed with anthracotic bronchitis are non-smoking elderly women living in a rural setting who have had no relevant occupational history for anthracosis(29, 30).

### Etiology:

The main cause of anthracosis/anthracofibrosis is unknown. However, there are several hypotheses that prolonged contact with biomass fuel emissions(2, 22, 31), genetics, chronic inflammatory reactions, air pollution, domestic pollution, and chronic infections such as tuberculosis (TB) play a role in the development of the condition(22). In this regard, there is controversy whether exposure to biomass fuels and previous history of TB should be considered etiologic factors.

Some researchers believe that anthracosis may be a predisposing factor for TB. In contrast, others believe that TB may lead to bronchial anthracosis without exposure to biomass fuel emission. Here, we review the literature for and against each theory:

According to various bacteriological and pathological evaluations as well as observed therapeutic responses, 27%–60% of anthracotic patients have been found to have TB (12, 17, 23, 32, 33). The typical presentation of TB in a patient with anthracofibrosis includes infrequent constitutional symptoms, rare granuloma formation, a negative purified protein derivative (PPD) test result, and low erythrocyte sedimentation rate (ESR). However, it has been unclear why these patients did not display characteristic manifestations of TB (7, 34). Two hypotheses were proposed to explain these atypical presentations. First, there may be a particular strain of *Mycobacterium tuberculosis* associated with this group of patients. In this regard, studies have shown that East-African-Indian superfamily (EAI) and Central-Asian superfamily (CAS) strains are frequent among anthracotic patients(30). The international spoligotyping database confirmed the high prevalence of these two strains in Middle Eastern countries, including Iran (35, 36). On the other hand, common TB subtypes in patients with anthracotic bronchitis were also noted, which leads us to believe the atypical manifestation of TB cannot be due to the presence of a specific strain of TB. The second hypothesis is based on the micro-environmental factors of *M. tuberculosis* growth. It has been shown that the hypoxic environment inside bronchial wall mucosa causes TB to grow slowly, which may lead to the immune system failing to properly recognize TB and a subsequent slighter response. Based on previous studies, the prevalence of the Beijing strain in the Iranian population is 3.2%; among Iranian multidrug-resistant TB patients, it is 20% (37). The Beijing strain is a rapidly growing strain (38); it causes active pulmonary TB and is not indolent. This may explain its low prevalence in patients with anthracotic bronchitis (30).

The rate of tuberculosis among patients with anthracosis differs in each province of Iran. It was 44% in Zahedan, 25%–30% in Mashhad, and 6.9% in Kerman. In contrast, there is no report of tuberculosis among patients with anthracosis from Tabriz or Sanandaj (39). In areas such as Tehran, Mashhad, Kerman and Zahedan, the prevalence of tuberculosis is relatively high, and simultaneous anthracosis is frequently reported from these regions. However, the correlation is premature because, until now, despite the high prevalence of tuberculosis in areas such as Khuzestan, Golestan, and Qom, anthracosis has not been reported (39, 40).

A close relation between tuberculosis and anthracofibrosis was described by Kim et al.(17) after evaluation of the images from 54 patients with anthracofibrosis, 32 of whom had a history of tuberculosis. This hypothesis was based on three signs: i) the association of active or old pulmonary tuberculosis with anthracofibrosis, ii) black anthracotic pigment formation during tuberculous treatment, and iii) similar imaging findings of both tuberculosis and anthracofibrosis. Bronchial narrowing, or atelectasis, was observed in most patients, with the right middle lobe (RML) bronchus being the most commonly involved. Conversely, the causative role of tuberculosis in anthracofibrosis, and, consequently, empiric tuberculosis treatment in patients with anthracofibrosis, was questioned by Park et al. (41). They evaluated 43 patients with anthracofibrosis and 32 patients with endobronchial tuberculosis who displayed bronchial stenosis on their computed tomography (CT) scans. However, they did not exclude those with a history of old tuberculosis from the group with anthracofibrosis. Seven of the 43 patients with anthracofibrosis had either active pulmonary tuberculosis or active tuberculous pleurisy. The authors found that, in contrast to endobronchial tuberculosis, anthracofibrosis was more common among elderly patients. Peribronchial and mediastinal lymphadenopathy, involvement of more lung lobes, bilateral lung involvement, and stenosis of any lobe of the right lung were significantly more common in anthracofibrosis patients than in endobronchial tuberculosis patients. It was also found that patients with endobronchial tuberculosis displayed contiguous luminal narrowing in the main and lobar bronchi, whereas the main bronchus tended to be unaffected in patients with anthracofibrosis. These differences between anthracofibrosis and endobronchial tuberculosis CT scan features suggest that tuberculosis may not be a causative factor in anthracofibrosis(42).

Anthracotic pigmentations are usually considered to be the result of carbon particle deposits, but iron, lead, cadmium, silica, phenol, hydrocarbon complexes, and other inorganic or organic substances may also cause this pigmentation. These pigmentations may be frequently observed in patients with no environmental exposure to coal dust or cigarette smoke(8, 9, 18). Exposure to biomass fuels was also suggested as one of the etiologies of anthracofibrosis. Kim et al.(18) studied 333 patients with anthracofibrosis, and found that all of them had a history of exposure to biomass smoke and presented clinical manifestations of obstructive airway disease. Such a high association to prior biomass exposure had not been reported elsewhere. Despite some suggestions of mixed-mineral dust toxicity being a contributing factor for anthracofibrosis, a study conducted by Mirsadraee and Saeedi(12) revealed no difference in the prevalence of dust exposure when comparing 41 patients with simple plaques of anthracosis to 22 cases of anthracofibrosis (42).

In conclusion, it seems that anthracofibrosis has an important relationship with pulmonary tuberculosis, either as a coexisting condition or a causative factor. Thus, the presence of TB should always be investigated when anthracofibrosis is present.

### Pathogenesis:

The exact pathogenesis of anthracofibrosis is still a mystery. There have been two hypotheses regarding clinical and histopathological findings. The first hypothesis is based on TB infection and explains the high coexistence of TB in anthracotic patients (2, 16, 31). The second theory was developed to describe why, in some studies, authors were unable to find any relation between TB and anthracofibrosis.

The first hypothesis theorizes that silica containing pigmentation causes alteration in the immune mechanisms of the lungs, making them prone to *M. tuberculosis* infection (43). Individuals with persistent long-term exposure to air pollutants, cigarette smoke, and biomass fuel smoke develop carbon and silica accumulation in their lymph nodes (44). If these lymph nodes become infected with *M. tuberculosis*, they rupture into the adjoining tracheobronchial tree, leading to black pigmentation, subsequent inflammation and fibrosis (6,10, 45). In favor of this hypothesis is the fact that, in some studies, anthracotic patients receiving tuberculosis treatment clinically improved (7).

The second hypothesis theorizes that biomass fuels (wood, manure, and harvest residues) are an important source for cooking and heating for about half of the world’s population, especially in rural areas of developing countries. These fuels generate carbon monoxide, nitrogen oxide, organic hydrocarbons, and other toxic compositions between 5 mm and 10 mm in size. Carbonaceous particles are deposited in two ways: 1) macrophages that are responsible for removing inhaled particles engulf the particles and remain in the submucosa (46); and 2) inhaled particles may remain in the bronchial tree due to deficient mucociliary clearance or abnormal macrophage activity, where they are then directly taken up by bronchial epithelial cells (47). The inhaled particles mostly accumulate at the branching portion of the bronchus, where there is a relatively lower clearance rate (48). Deposited carbonaceous particles cause fibrosis of the bronchial wall or the surrounding interstitium, resulting in hypertrophy of the bronchial wall and narrowing of the bronchial lumina (18). It seems that neither of these hypotheses can explain every aspect of anthracofibrosis pathogenesis. Therefore, there may be two possible causes of the same entity (16).

### Clinical Manifestation:

Most patients with anthracosis present with dypnea (90% – 100% of cases) and cough (in 29.8% – 83.6% of cases) (15). Hemoptysis, or non-specific chest pain, constitutional symptoms, and sputum production – both black and watery – are observed less frequently. These symptoms vary in frequency between studies (10, 19, 21, 49, 50). New onset weight loss or fever (51), enlarged mediastinal lymph nodes, and subsequent complications such as vocal cord paralysis (52) or broncholithiasis (45) may be the symptoms initially observed in anthracofibrosis patients. The most common symptom associated with pulmonary auscultation in patients with anthracosis is wheezing (7); rales or decreased breathing sounds are noted less frequently (32). Normal physical examination is noted in some cases but the exact prevalence is unclear (19). One possible explanation for this variation may be the association of anthracofibrosis with other diseases, resulting in altered clinical features for some patients.

### Natural course:

The course of anthracofibrosis is chronic, and it is commonly misdiagnosed as chronic bronchitis. Most patients with bronchial anthracotic lesions had a stationary and inactive course that might be interrupted by acute attacks (20).

When a known case of anthracofibrosis is not responding to conventional therapy or is having an unexpectedly poor clinical course, additional clinical evaluation is performed using bronchoscopy or chest CT scans (53). Pulmonary infection, including tuberculosis and pneumonia, and exacerbation of obstructive airway disease are the most commonly associated diseases presenting with persistent localized wheezing or abnormal chest X-ray (CXR) findings with or without respiratory symptoms. The researchers suggest that pneumonia or acute exacerbation of obstructive airway disease may occur repeatedly during the clinical course of bronchial anthracofibrosis (18, 25).

### Associated conditions:

Although some studies report a strong association between anthracofibrosis and TB, Park et al. (41) does not. We assume this discordance originates from the populations used in each study; if a study consists of patients from TB endemic regions, it may show no association. Alteration of the pulmonary immune defense mechanisms due to toxic substances in wood smoke (54), increased sensitivity to *M. tuberculosis* due to silica containing pigmentation (43), the prevalence of TB increasing with age (55), and an increased risk of TB with exposure to biomass-fuel smoke may all be considered reasons to confirm the association.

Currently, exposure to biomass-fuel smoke is considered a risk factor for both COPD and anthracofibrosis, and the association between the two seems to be inherent. Previous studies have also reported a high prevalence of pneumonia in patients with anthracofibrosis, especially those with no clinicopathological features of TB (56, 57). Structural abnormalities caused by exposure to biomass fuel and consequent consolidation, which is seen in lobes with bronchial narrowing, along with changes in the defense mechanisms of the lung mucociliary system and a decrease in the antibacterial capacity of macrophages, are known to be contributing factors for the development of pneumonia in these patients(57, 58).

Exposure to biomass fuel results in a lower peak expiratory flow rate and significant bronchial hyperresponsiveness. Thus, it is common to find asthmatic clinical characteristics in patients with anthracofibrosis or worsened symptoms of bronchial asthma in previously diagnosed patients. Kim et al.(18) reported that 19 of 333 patients developed asthma, and Lee et al. (24) reported that two of 46 patients did. Gupta and Shah also emphasized the need for further investigation regarding the clinical course of anthracofibrosis patients with and without asthma (15).

A significant correlation between wood-smoke exposure and sarcoidosis was first described by Kajdasz et al (59). In another study by Kreider et al. (60), wood-smoke and organic-dust exposure were shown to correlate with sarcoidosis. Gunbatar et al. also reported four patients with at least 29 years of biomass exposure and a mean age of 53.25 years showing subcarinal enlargement; they were diagnosed with sarcoidosis and were responsive to steroids (58).

### Mediastinal lymphadenopathy:

There are several causes of mediastinal lymphadenopathy, including infection, neoplasia, and granulomatous disease. In developing countries, anthracosis should also be considered a cause of mediastinal lymphadenopathy(61). Lymphadenopathy caused by anthracosis is usually intrapulmonary, but, rarely, it may also present as a mediastinal mass, mediastinal lymphadenopathy, or axillary lymphadenopathy. Moreover, intrathoracic lymph nodes are often anthracotic in elderly persons(15).

Kirchner et al. confirmed involvement of mediastinal lymph nodes with Endobronchial ultrasound-guided transbronchial needle aspiration (EBUS-TBNA) and then analyzed their anatomical position using multi-slice computed tomography. They found that the most common site of anthracotic lymph nodes was the subcarinal area (62). Yilmaz Demirci et al. also reported subcarinal and interlobar lymph nodes to be the most commonly affected stations (36.8% and 34.3%, respectively). It has been proposed that anthracotic mediastinal lymph nodes are most often a well-defined oval shape, with frequent calcifications (61).

### Cancer susceptibility:

Cigarette smoke is a definitive cause of lung cancer. This effect is caused via induction of p53 gene mutations by benzapyrene (BaP) and nicotine-derived nitrosamine ketone (NNK), which are among the 60 carcinogenic components of cigarettes. Wood smoke contains BaP as well (63). Delgado et al. detected a mutation in the p53 gene in lung cancer patients who were exposed to wood smoke or who were cigarette smokers. They proposed that, just like cigarette smoke, wood smoke can play a role in the development of lung cancer (64).

Malats et al. noted a higher frequency of genetic polymorphism in glutathione S-transferase enzymes in non-smoking lung-cancer patients who were exposed to wood smoke for more than 20 years (65). Liang et al. (66) investigated the effects of exposure to coal and wood smoke in mice and rats in regions of China where lung cancer is common in humans and found that wood-smoke exposure involved a higher risk of cancer than the control, but a lower risk compared to coal-smoke exposure. Gunbatar et al. (58) has also shown that biomass-smoke exposure is an important risk factor in human lung cancer. Gold et al. (27) proposed that cooking in a poorly ventilated atmosphere may produce high levels of BaP, equal to that in packs of cigarettes a day. In another study, BaP levels detected over the course of three hours of cooking a day was the same as two packs of cigarettes smoked a day. These estimations facilitate the hypothesis that biomass-smoke exposure can contribute to the growth of malignancy. Adenocarcinoma showed to be the most prevalent histopathological type of lung cancer in non-smoking women exposed to wood smoke. Similarly, in a study conducted by Delgado et al. (64), the main histopathological type was adenosarcoma in patients exposed to wood smoke. On the other hand, small-cell carcinoma was the primary type of lung cancer in the study conducted by Gunbatar et al (58). The reason for this controversy in regard to histological subtype prevalence may be the different and specific composition of the biomass in each region, as it might contain a different composition of carcinogens (58). Hoffmann et al. performed a series of investigations on lab animals and concluded that NNK was associated with adenocarcinoma, and BaP was associated with squamous-cell carcinoma (67).

### Bronchopneumonia susceptibility:

It could be that bronchopneumonia is prevalent in anthracotic patients due to bronchial narrowing, which leads to poor drainage of secretions and predisposes the patient to recurrent infections (68), but Singh et al. did not find a significant association between bronchopneumonia and anthracosis in their study population (10).

On the other hand, Cho et al. reported a high incidence of pneumonia in up to 40% of anthracotic patients and opined that the location of pneumonic consolidation on a CXR is often consistent with lobes that have bronchial narrowing. Thus, bronchial anthracofibrosis may be a major risk factor for pneumonia (69).

### Additional organ involvement:

Anthracosis may affect other organs in the body, as there are reports of liver and spleen involvement. As calcification is a radiographic sign of the disease, Mirsadraee et al. have recommended considering anthracosis among the possible diagnoses when presented with high-attenuation images from all parts of the body. The diagnosis should be confirmed using histopathological evaluation(13).

### Concomitant esophageal anthracosis:

Esophageal anthracosis is a rare disease. Yang et al. reported esophageal anthracosis occurring simultaneously with endobronchial anthracosis. The underlying mechanism of this coexistence is unclear. There have been two hypotheses presented in literature: First, peribronchial lymph nodes become inflamed due to anthracotic particles extending toward the esophagus and the traction diverticula, and black-pigmented mucosal changes consequently arise. Second, inadvertent ingestion of a substance containing coal causes deposits in previously undiagnosed esophageal ulcerations. Considering the rarity of this entity, there is no definite information regarding the prognosis or treatment of esophageal anthracosis. However, it seems that histological confirmation should be conducted to exclude the diagnosis of malignant melanoma of the esophagus, even though endoscopic characteristics are compatible with benign lesions (70).

### Lung parenchymal involvement:

In some cases, anthracofibrosis may involve lung parenchyma with the same mechanism involving the bronchial tree. Lung parenchyma involvement includes the deposit of anthracotic pigmentation with focal fibrotic lesions without granuloma formation or infection by pathogens (7). For this reason, Yoon et al. stated that anthracofibrosis involving lung parenchyma is a peripheral focal lung lesion that appears as a nodule, mass, or fibrotic consolidation. The study followed 34 patients for a mean period of 790 days, and 33 showed no significant changes in serial CXR during follow-up examinations. Given the long-term stability of anthracofibrosis involving lung parenchyma, it may be considered by some authors to be a benign lesion that does not require further treatment such as anti-tuberculosis or antibiotic medications(5).

### Diagnosis:

The definitive diagnosis of anthracofibrosis is usually achieved during a bronchoscopic examination of patients with chronic cough, sputum, and dyspnea (9).

Pulmonologists should consider the possibility of this disease, especially in older, non-smoking women exposed to biomass combustion products. Sputum samples should be taken for acid-fast bacilli smear and culture, and, if TB is confirmed, the proper treatment with radiographic follow-up should be initiated (28).

### Pathology:

Amoli (20), Gunbatar et al. (58), Restrepo et al. (71,) and Ramage et al. (72) discussed pathological findings in anthracofibrosis, and have reported almost the same characteristics. Histopathological findings in biopsy samples of anthracofibrosis patients revealed infiltration of non-specific mononuclear inflammatory cells, mostly macrophages. Polymorphonuclears, eosinophils, lymphocytes, and plasma cells both in the epithelium and stroma were also present, forming edema and granulation tissue (58). The epithelium, however, was intact and the pseudostratified arrangement with ciliated and goblet cells preserved. Only occasional squamous metaplasia and dysplasia were noted. Congestion, hyper vascularization, thickening of the basement membrane and fibrosis were also reported. The main pathological changes in the transbronchial tissue samples of patients exposed to inorganic dust were the intracellular and extracellular black particles observed in the epithelium and stroma (58). Other pathological lesions may be differentiated as cigarette-induced chronic bronchitis, which is characterized by the proliferation of goblet cells, hypertrophy of submucosal glands, an increase of the Reid index, epithelial dysplasia, and a tendency toward neoplasia. Hypersensitivity pneumonitis is also characterized by granuloma formation. However, pathological specimens in anthracofibrosis were not indicative of granulomas (20).

### Respiratory function tests

Information on pulmonary function test alterations in anthracofibrosis is limited. Amoli used spirometry to investigate 102 anthracotic patients; he reported approximately two-thirds of the patients to show obstructive patterns, while one-third manifested restrictive patterns (20). The Korean studies have reported the obstructive pattern to be the most frequent, but the frequency of normal and restrictive patterns was higher than what was reported in Amoli’s study (56, 57). Mirsadraee et al. also assessed pulmonary function using static and dynamic spirometry in two equal groups of 40: an anthracofibrosis group and a control group. They reported dyspnea, cough and wheezing as the most common symptoms in their cases. They also reported a significant decrease in all parameters including VC (FVC), FEV1, FEV1/FVC, FEF25-75 and FEF25-75 /FVC. Along with previous studies, Mirsadraee found obstructive patterns in 95% of subjects based on a low value of FEV1/FVC and FEF25-75 and the increment of RV in 95% of their study group. In that study, the diffusing capacity of the lungs was reported to be normal in all patients, which ruled out emphysema. Authors rationalized that the high frequency of obstructive pattern in their study to be due to the inclusion criteria set for patient enrollment, as they allowed entrance of patients only with anthracofibrosis. The Korean studies and Amoli’s study, on the other hand, enrolled every subject with black discoloration and without bronchial deformity (14, 20, 56, 57).

### Radiology:

Radiological abnormalities are discovered in the CXRs and CT scans of almost all anthracosis and anthracofibrosis cases. Recently, a few studies reported that atelectasis, along with mediastinal lymph-node calcification, is a radiological sign for anthracofibrosis, but a main limitation to these studies was the lack of a control group for statistical analysis (13).

Mirsadraee et al. tried to overcome this limitation by performing a prospective, case-control study including three groups of 70 patients with a bronchoscopic diagnosis of simple anthracosis and anthracofibrosis and 40 patients with a non-anthracotic diagnosis as control group. In this study, in both simple anthracosis and anthracofibrosis, lymph-node and bronchial calcifications proved to be the most significant radiological findings. The odds ratio of lymph-node calcification was 22.9 in anthracosis, with 83% sensitivity and 89% specificity. They also reported bronchial calcification as a useful finding, as it had an odds ratio of 9.4 for anthracofibrosis (13).

In this regard, the third most-common radiological finding in anthracosis/anthracofibrosis patients was a benign mass with or without calcification. Other radiological findings noted at a lower frequency included collapse, bronchial stenosis, and collapse consolidation (13). Another study from Iran by Amoli (20) documented abnormal CXRs of anthracosis patients with streaky shadows along the broncho-vascular marking in the paracardiac and parahilar regions. Törün et al. (8) reported the same finding in CXR and CT imaging, but with different frequencies; in their study, collapse was detected in 48% of patients, whereas linear (streaky) shadows were detected in 40% of patients.

The predominant site of involvement was reported to be the RML in studies by Chung et al. (7), Hemmati et al. (73), and Kim et al(17). However, in studies by Mirsadraee et al. (13), Touhidi et al. (51), Törün et al. (8), and Kim et al.(18), the upper lobe was the predominant site of involvement.

Kim et al. (18) reported CT scan findings of anthracofibrosis with bronchial narrowing and atelectasis with smooth narrowing as the most common radiological findings in 94% and 80% of patients, respectively. In their study, 63% of patients developed enlarged lymph nodes; among those, 57% were calcified.

There are two main diagnoses for these CT findings: bronchogenic carcinoma and endobronchial tuberculosis, which should be distinguished from anthracofibrosis. Park et al.(41) defined specific characteristics of each. Bronchogenic carcinoma involves focal areas of a single lobe or segment of bronchus, usually in the form of endobronchial nodular projections.

Identifying bronchostenosis caused by anthracofibrosis may be challenging, and this may be the reason many authors disagree regarding the causative relation between anthracofibrosis and TB. However, bronchostenosis in anthracofibrosis is multifocal and has segmental or lobar bronchi involvement in both lungs. The main bronchus and trachea are intact, and there is no relationship between the extent of the disease and the radiological features. On the other hand, TB develops a bronchostenosis that appears in a single lobar bronchus in a continuous spreading pattern. Luminal narrowing of the ipsilateral main bronchus and distal trachea is common. These differences may be evidence against the hypothesis which named TB as a major causative factor for anthracofibrosis (41).

### Fludeoxyglucose Positron Emission Tomography (FDG PET):

It has been reported that Positron Emission Tomography (PET) has greater than 90% sensitivity, but a specificity of only about 80% in diagnosing pulmonary pathology. Fludeoxyglucose (FDG) is a glucose analogue that is transported into both normal and malignant cells. Thus, it may be accumulated in inflammatory cells such as neutrophils and activated macrophages at the site of inflammation or infection, or it may be accumulated in active granulomatous processes such as tuberculosis and sarcoidosis, causing false positive PET-scan results for malignancy. Anthracotic particles can also cause antigenic stimulation of macrophages. Hence, increased FDG uptake has been reported on PET scans of anthracotic lymph nodes in the neck, hilar region, and mediastinum and anthracotic pulmonary nodules. In a report of mediastinal lymphadenopathy associated with anthracosis and exposure to wood smoke, the nodes were metabolically active on PET/CT scans and the mean maximum standardized uptake value (SUVmax) within the lymph nodes was from 5 to 8.4. In the study conducted by Yilmaz Demirci et al., 201 lymph nodes were sampled from 106 patients with a mean duration of exposure to biomass of 35.5 years. The reported SUVmax value within the lymph nodes was 4.76 (1–16.8) and was indistinguishable from malignant or granulomatous conditions (61).

In conclusion, the possibility of benign conditions should be considered when intense uptake is observed on a PET scan in hilar and mediastinal lymphadenopathy, especially when there is a history of exposure to known risk factors (29).

### Bronchoscopy:

Currently, the diagnosis of anthracosis is mostly made via bronchoscopy because other non-invasive diagnostic procedures are not very accurate in identifying the disease(13).

The principal findings in cases of bronchial anthracofibrosis include multiple pigmented anthracotic lesions and bronchial stenosis. The mucosa around the branching points of the bronchus is the most frequent anatomical site for anthracosis. Although bronchoscopic findings may vary in different countries, the majority of them are indicative of involvement of both upper lobes and the Right middle lobe (RML). Korean studies reported right upper lobe (RUL) involvement in 302 of 333 patients (90.7%), left upper lobe (LUL) involvement in 289 (86.8%), and RML involvement in 229 (68.8%) (18). A study from Turkey also described RUL involvement in 25 of 27 patients (92.6%) and RML involvement in 22 (81.5%) (8).

Bronchial narrowing was most commonly observed at the level of the lobar and segmental bronchus, particularly in the RUL and RML bronchi. RUL bronchus narrowing was seen in 213 of 333 (64%) patients from Korea and in 14 of 27 (51.8%) patients from Turkey. In both studies, the majority of patients had multifocal narrowing, with more than one part of the bronchial tree narrowed (8,18).

Bronchial stenosis associated with endobronchial TB has also been observed, with the RML bronchus most commonly involved. Brock et al. described this finding based on middle lobe syndrome and assumed that the strategic location of the middle lobe bronchus in relation to the bronchus intermedius was responsible for the middle lobe atelectasis (74).

### Endobronchial ultrasound-guided transbronchial needle aspiration (EBUS-TBNA):

Endobronchial ultrasound-guided transbronchial needle aspiration (EBUS-TBNA) is a minimally invasive procedure performed under local anesthesia allowing for real-time assessment and biopsy of mediastinal lymph nodes. Therefore, it would negate the need for mediastinoscopy. It has a sensitivity, specificity and accuracy of 95.7%, 100%, and 97.1%, respectively, in distinguishing benign from malignant mediastinal and/or hilar lymph nodes. When a sample is taken with EBUS-TBNA, the reintroduction of the stylet into the needle creates friction between the two surfaces and results in the release of metal particles. These particles are always clearly visible, extracellular, and differing from dusts or anthracosis. Anthracosis is the most frequently endogenous source of particles in mediastinal lymph node samples, and is mostly located inside macrophages. Anthracotic particles have a different origin than alveolar macrophages engulfed by phagocyte cells, as they have been previously inhaled, then transported by the lymphatic system to the nodes. This is in contrast with particles that may detach from the needle. Thus, EBUS-TBNA could play an important role in defining observed particles (61).

### Diagnosis of TB in anthracosis patients:

Clinical symptoms such as cough, hemoptysis, and even radiological characteristics of tuberculosis such as upper-lobe localization and cavity formation were not observed more frequently in anthracosis patients suffering from TB. However, laboratory tests including smear for acid fast bacilli (AFB), culture and histopathological findings such as caseating granuloma were successful in diagnosing TB. Thus, diagnosis of TB in anthracotic patients should be based on these findings. However, these diagnostic modalities are reported to have different accuracies. Touhidi et al. (51) detected TB by direct smear for AFB in 96%, and Törün et al. (8) detected TB by culture in 100%. On the other hand, Hemmati et al. (73) and Kim et al.(18) diagnosed TB by biopsy and histopathological evaluation in 100% and 83% of their subjects, respectively. Ghanei et al.(4) reported cavitary lesion, a positive PPD test, and a high sedimentation rate as the most helpful ancillary paraclinical indications for the diagnosis of TB in anthracosis patients.

PCR (polymerase chain reaction) enabled the diagnosis of TB better than traditional tests, but the difference between PCR and the accumulation of traditional methods was not significant. Therefore, a complete profile of traditional TB tests, including AFB, culture, and histopathology, provided results comparable to those obtained by PCR in BAF patients (68).

### Prevention and Treatment:

There is no specific treatment for anthracofibrosis, and most of the available treatments, including antibiotics for concomitant respiratory infections, bronchodilators, mucolytic agents and inhaled corticosteroids, provide symptomatic relief with no effect on the underlying pathogenesis. However, previous studies have suggested that there may be some value in empirically treating such patients for tuberculosis. So far, there have been no controlled trials to evaluate the efficacy of treatment with anti-tuberculosis drugs, but promising results have been reported.

Tutluer et al. believed empirical anti-tuberculosis treatment may be reasonable for patients with anthracofibrosis who live in areas where TB is endemic, and that it may lead to definite improvement of chest radiograph findings. They also brought attention to the fact that, even in developed countries, immigrants may be presenting with anthracosis and/or TB(1). Boonsarngsuk et al.(9) used corticosteroids and tamoxifen based on studies by Meredith et al. (75) and Clark et al. (76), which showed positive effects of a combined course of corticosteroids and tamoxifen in the treatment of idiopathic mediastinal fibrosis. Treatment was initiated with prednisolone at a starting dose of 40 mg/day and tamoxifen at 20 mg/day for four weeks. Tamoxifen was then continued while prednisolone was tapered, which resulted in clinical and radiological improvements (9). In contrast, Thrumurthy et al.(77) reported no clear benefits of steroid therapy, and stated it may lead the doctor to anxiety when treating a patient with TB. Bircan et al. treated a Turkish anthracofibrosis patient with anti-tuberculosis medications along with corticosteroid therapy in tapering doses. However, the patient died during follow-up due to gastric cancer, preventing the determination of a long-term prognosis (52).

The other field of treatment has been introduced by El Raouf et al. They rationalized that mechanical dilation or endobronchial stents, which are commonly used in patients with other malignant and non-malignant airway disorders, could be used in patients with endobronchial anthracofibrosis. They described two cases with severe symptomatic bronchial stenosis resulting from anthracofibrosis. Both patients responded to endobronchial stent placement, which provided excellent relief of symptoms (78). Further investigation, especially in the form of randomized clinical trials, is required to establish a definitive treatment for anthracofibrosis.

### Cases of misdiagnosed anthracotic lymph node with lung metastasis:

There were a few reports in the literature regarding the possible benign differential diagnosis of intrapulmonary round lesions, especially in patients with previous cancer history. This type of lesion has characteristic radiological features, including a sharp margin with a homogenous inner pattern. Here we represent two cases.

A 58-year-old male with a history of bladder and prostate cancer who had multiple typical round lesions on a chest-computed tomography was admitted for further evaluation. The most probable diagnosis was metastatic disease of the lung, but wedge-resected specimens were compatible with anthracotic lymph nodes (79).

The authors used PET scans with radiolabeled choline (18F-Fcholine) during the pretreatment stage of a high-risk prostate adenocarcinoma in a 62-year-old male. A significant mediastinal lymph node uptake of 18F-FCholine was noted. Considering that the spread to supradiaphragmatic lymph nodes is rare, surgical removal was performed, and no malignant cells other than those with anthracosis were revealed (80).

These reports suggest that benign lesions such as anthracotic lymph nodes, although less common, should also be considered as differential diagnoses of intrapulmonary round lesions in patient with known preceding malignant disease. However, false positives of radiological modalities should be noted as well. Thus, histological assessment should be performed in order to decipher the appropriate treatment.

## CONCLUSION

In light of our findings, it seems that previously known anthracotic pigmentation in the bronchial tree is a manifestation of a greater syndrome. This histopathological finding may be observed in other parts of both respiratory and non-respiratory organs sharing the same pathogenesis, leading to almost the same clinical picture. In this regard, we suggest that it is better to consider all different aspects of anthracosis as ANTHRACOSIS SYNDROME. This comprehensive attitude may be helpful to 1) better understand what we are dealing with, 2) estimate its importance, especially in association with TB, 3) find potential cancer susceptibility, and 4) provide rapid and safe treatment whenever indicated.
